# An Investigation into the Quality of Medicines in Yangon, Myanmar

**DOI:** 10.3390/pharmacy6030096

**Published:** 2018-08-30

**Authors:** Md. Rafiqul Islam, Naoko Yoshida, Kazuko Kimura, Chisana Uwatoko, Mohammad Sofiqur Rahman, Shoma Kumada, Jamie Endo, Kana Ito, Tsuyoshi Tanimoto, Theingi Zin, Hirohito Tsuboi

**Affiliations:** 1 Institute of Medical, Pharmaceutical and Health Sciences, Kanazawa University, Kanazawa 920-1192, Japan; rafiqbgeiu@gmail.com (M.R.I.); naoko@p.kanazawa-u.ac.jp (N.Y.); kimurak@p.kanazawa-u.ac.jp (K.K.); Morahmansofique@gmail.com (M.S.R.); dmpc11@p.kanazawa-u.ac.jp (S.K.); dmpc13@p.kanazawa-u.ac.jp (J.E.); 2 Department of Analytical Chemistry, Faculty of Pharmaceutical Science, Doshisha Women’s College, Kyoto 610-0395, Japan; cuwatoko@dwc.doshisha.ac.jp (C.U.); yke043@dwc.doshisha.ac.jp (K.I.); tsuyoshi-tanimoto@pmrj.jp (T.T.); 3 Department of Food and Drug Administration, Naypyidaw 15000, Myanmar; zintheingi9@gmail.com

**Keywords:** poor quality medicine, substandard medicine, counterfeit medicine

## Abstract

Many poor-quality medicines are supplied to patients mainly in developing countries. No systematic survey on counterfeit medicines has been conducted in Myanmar since 1999. The purpose of this study was to investigate the current situation of substandard or counterfeit medicines in Myanmar. Samples of oral medicines, cefuroxime axetil (CXM), donepezil hydrochloride (DN) and omeprazole (OM), and injections, ceftriaxone sodium (CTRX), and gentamicin sulfate (GM), were collected from pharmacies, hospitals, and wholesalers in Yangon, Myanmar in 2014. Authenticity and quality were verified. There were 221 (94%) foreign medicines among 235 collected samples. Five samples of GM and 1 DN sample were not registered with the Food and Drug Administration, Myanmar. In quality analysis, 36 samples out of 177 (20.3%) did not pass quantity tests, 27 samples out of 176 (15.3%) did not pass content uniformity tests, and 23 out of 128 samples (18.0%) did not pass dissolution tests. Three of the unregistered GM samples failed in both identification and microbial assay tests. Counterfeit GM is being sold in Yangon. Also, the quality of OM is a matter of concern. Poor-quality medicines were frequently found among the products of a few manufacturers. Regular surveys to monitor counterfeit and substandard medicines in Myanmar are recommended.

## 1. Introduction

Medicines are essential for human health, but many substandard or counterfeit medicines are supplied to patients [1,2], and in this vast sector it is very difficult to identify these products and develop effective preventive measures. We have been attempting to clarify distribution channels of such poor-quality medicines, and to check the spread of such medicines. Although the World Health Organization (WHO) reported that the proportion of substandard or counterfeit drugs supplied in developed countries was approximately 1% [3], the risks appear to be increasing according to the development of the Internet and the progress of globalization [4]. WHO also reported that the proportion of such poor-quality drugs supplied in developing countries was about 10% [3]. It is significant to investigate the conditions of substandard or counterfeit medicines in developing countries for preventing health damages by poor-quality medicines. In a study of 104 samples of anti-malarials in Southeast Asia in 2001, 38% were found to be substandard or counterfeit [5]. In Cambodia in 1999, substandard or counterfeit artesunate containing sulfadoxine-pyrimethamine caused the death of at least 30 people [6]. The incidence of substandard or counterfeit medicines was estimated to be in the range of 4%–90% from 2001 to 2010 in Cambodia [7,8,9]. In China in 1999, 2 people died after taking anti-diabetic drugs that contained illicit glibenclamide, while in Singapore in 2008, 4 people died after taking substandard or counterfeit phosphodiesterase type 5 inhibitor containing glibenclamide [5,10]. In India, 30 infants died in 1998 after receiving substandard or counterfeit paracetamol [11]. Around 200 children died in Bangladesh in 1990–1993 after ingesting substandard or counterfeit paracetamol that contained diethylene glycol [12]. In Myanmar, one man aged 23 died in 2005 after taking substandard or counterfeit artesunate [13]. Furthermore, a massive investigation by WHO in 1999 identified various substandard or counterfeit medicines in Myanmar [14].

Medicines that are substandard or counterfeit can include poor-quality medicines that are below the standards on the label for various reasons, such as degradation [15]. Substandard medicines are genuine medicines produced by legitimate manufacturers that do not meet the quality specifications declared by the producer [16]. Degraded products may result from exposure of good-quality medicines to light, heat, and humidity; however, it can be difficult to distinguish degraded medicines from those that left the factory in substandard condition [17]. Substandard and degraded medicines can reduce the effectiveness of therapy.

The aim of this study is to investigate the current situation of substandard or counterfeit medicine in Myanmar to provide baseline data for preventing the spread of such medicines in the future.

## 2. Materials and Methods

### 2.1. Sample Collection

Sampling was carried out in Yangon, a former capital of Myanmar with a population of nearly six million. Samples of oral medicines, cefuroxime axetil (CXM), donepezil hydrochloride (DN) and omeprazole (OM), and injections, and ceftriaxone sodium (CTRX) and gentamicin sulfate (GM), were collected from 27 September to 4 October 2014 by two teams (Table 1). Each team contained one supervisor from Food and Drug Administration, Myanmar (MFDA), one local assistant, and one or two Japanese researchers. All team members had received training before starting the sampling work. Samples were collected from a government hospital and a private hospital designated by the MFDA, as well as community pharmacies, clinical pharmacies, and wholesalers nearby. Sampling forms were maintained and purchased samples were stored at 20–25 °C before being transferred to Kanazawa University for analysis.

### 2.2. Observation Test

The samples were observed according to “Tool for Visual Inspection of Medicines” produced by the International Council of Nurses in partnership with the United States Pharmacopoeia (USP) and modified by the Military and Emergency Pharmacists Section of International Pharmaceutical Federation (FIP) [18]. Samples were checked for 48 points. During sampling, we also observed the conditions of the outlets, including temperature and hygiene, and photographs were taken for the record. Spelling on labels, MFDA registration number, and drug insert details were checked at Kanazawa University.

### 2.3. Authenticity Investigation

An authenticity investigation was performed according to the modified WHO method [19]. The procedure is to ask the manufacturers stated on the label of the product about authenticity and also to ask the Medicines Regulatory Authorities (MRA) of the manufacturing countries about the legitimacy of the products and manufacturers. Questionnaires, including pictures of the sample and (if requested) some tablets, were sent to the manufacturer and MRA for evaluation of authenticity. Registration numbers in Myanmar were verified by the MFDA and Ministry of Health, Myanmar (MMOH).

### 2.4. Quality Test

Test methods were established for each medicine according to the pharmacopoeia cited on the packaging of the medicines, i.e., USP, British Pharmacopoeia (BP), or Japanese Pharmacopoeia (JP). Identification, quantity test (QTY), content uniformity test (CU), and dissolution test (DS) were conducted for tablets and capsules of CXM, DN, OM, QTY, and CU. Identification, bacterial endotoxin, and sterility tests were performed for CTRX injection. Identification, bacterial endotoxin, sterility, and microbial assay tests were performed for GM injection.

### 2.5. Statistical Analysis

Data were analyzed using the IBM SPSS Statistics version 24 (IBM Japan, Tokyo, Japan). Fisher’s exact test was performed to evaluate the significance of differences in categorical variables, and the *t* test for continuous data. The criterion of statistical significance was set at the level of 5%. 

### 2.6. Ethics Approval

This study protocol was approved by and got permission from MFDA.

## 3. Results

### 3.1. Sample Collection: Province and Outlet Type

The results of sample collection are summarized in Table 1. A total of 235 samples, consisting of CXM tablets (n = 60), DN tablets (n = 3), OM capsules (2 samples for tablet) (n = 65), CTRX for injection (n = 49), and GM injections (n = 58) were purchased from 75 retail shops. We obtained 103 samples from community pharmacies, 47 samples from the government hospital, 42 samples from the private hospital, 28 samples from clinical pharmacies, and 15 samples from five different wholesalers.

### 3.2. Environmental Conditions in Pharmaceutical Shops

All shops were equipped with awnings to protect medicines from direct sunlight. On the other hand, only 29 of 75 shops (39.2%) were equipped with air conditioning. The average temperature in shops without air-conditioning was 30.8 ± 2.16 °C, while it was 28.6 ± 2.64 °C in shops with air-conditioning (*t* test, *p* < 0.01). The average humidity in shops without air-conditioning was 69.3 ± 8.70%, while it was 67.9 ± 12.37% in those with air-conditioning (*t* test, not significant). In some cases, motorbikes, dogs, or cats were observed in the dispensing area of shops. These conditions are potentially insanitary in a medical facility.

### 3.3. Sample Forms and Number of Manufacturers

Table 2 lists the sample forms and countries from which the samples were imported. The strengths and forms were 1 g powder for injection in a vial for CTRX, 250 mg tablets for CXM, 5 mg tablets for DN, 80 mg/2 mL injections in ampoules for GM, and 20 mg tablets and 20 mg capsules for OM.

The total number of manufacturers was 71, among which 8 offered different brands of the medicines. The numbers of manufacturers were 19 for CTRX for injection, 12 for CXM tablets, 2 for DN tablets, 17 for GM ampoules, and 21 for OM tablets and capsules. Fourteen samples out of 235 (6%) were produced by 4 manufacturers in Myanmar, while 221 (94%) samples from 59 manufacturers were imported from 12 countries. Among the latter, 150 samples out of 235 (63.8%) were imported from India, and 17 (7.23%) from China.

### 3.4. Observation Test

Concerning storage conditions, 29 out of 75 retail shops (38.7%) had air-conditioning. Information, trade names, or manufacturers’ name did not match among inserts, containers, blisters, or home pages in several cases, that is, there was no batch number on one blister of CXM, the manufacturer’s address was different on the box and on the insert of two CXM samples, and no registration number was attached on one blister of CXM. There were spelling mistakes in the English on the back of the package boxes of 11 samples of CXM and 2 samples of GM.

### 3.5. Authenticity Investigation

We sent questionnaires to 63 manufacturers of CXM, DN, OM, CTRX, and GM by email in October 2014, and 6 manufacturers have replied so far. We also sent questionnaires to 12 MRAs in manufacturing countries other than Japan (for Japan, we directly checked official documents) by email, and relevant information was obtained from MRAs in Myanmar and Switzerland. The Bangladeshi MRA notified us that they had received the letter, but did not answer the questionnaire.

It was found that all samples of CTRX, CXM, and OM were registered, whereas 1 DN sample out of 3 and 5 GM samples out of 58 were not registered by MFDA (Table 3). The 1 unregistered DN sample was of Indian origin. Of the 5 unregistered GM samples, 3 samples (from 2 companies) were of Chinese origin and 2 were of Indian origin. The 2 Chinese companies were not registered by MFDA. Also, the GM samples from India were not registered. One Indian company replied to our inquiry, stating that their product is “Not for export”.

### 3.6. Quality Analysis

**Figure 1:**
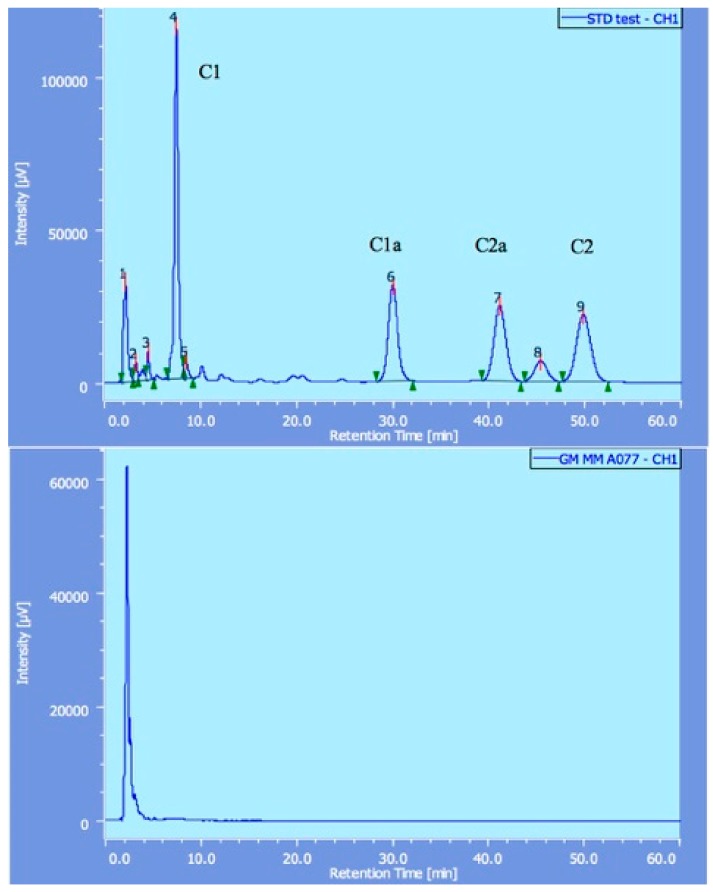
The results of gentamicin detected by high performance liquid chromatography (HPLC). Standard gentamicin showed four peaks (C1, C1a, C2 and C2a in the upper diagram), whereas counterfeit gentamicin did not show any of these peaks (lower illustration).

Table 4 presents the results of the quality tests. Out of 177 samples, 36 (20.3%) did not pass the quantity tests. In content uniformity tests, 27 samples among 176 (15.3%) did not pass, while 23 out of 128 samples (18.0%) did not pass the dissolution tests. In the endotoxin and sterility tests, all the sampled medicines (CTRX and GM) passed (100%). For ampoules (GM), we also performed identification and microbial assay examinations. Three unregistered GM samples out of 58 failed in identification, as shown in Figure 1; they showed no peaks, which means that these samples do not contain any effective ingredient. In addition, these 3 samples did not pass the microbial assay tests. The Myanmar Government announced that the GM ampoules from two Chinese manufacturers (3 samples) were counterfeit. CXM samples that did not pass the quality tests originated from a certain Indian manufacturer, whereas unacceptable OM samples were not from particular manufacturers.

### 3.7. Prices

The average prices of all-exam-passed samples of CXM were significantly higher than those of other samples (unpaired *t* test: *p* < 0.05), whereas OM did not show any significant differences between all-exam-passed samples and others (Table 5). The prices of counterfeit GM samples appeared to be lower in comparison with good-quality samples (Table 5).

## 4. Discussion

We investigated the current situation regarding substandard or counterfeit medicines in Yangon, Myanmar, by collecting CXM tablets, DN tablets, OM tablets/capsules, CTRX for injection, and GM for injection from pharmacies, a governmental hospital, a private hospital, clinics, and wholesalers. Substandard or counterfeit medicines with little or no potency were identified, and there were some unregistered products among GM and DN. Regarding quality, we found poor quantity and poor content uniformity in some samples of CTRX, CXM, and OM, as well as poor dissolution in some samples of CXM and OM, and some samples of GM that contained no effective ingredient. 

Resistance to third-generation cephalosporin series and aminoglycoside series compounds has been documented globally. Especially, resistance of *Klebsiella pneumoniae* and *Neisseria gonorrhoeae* to third-generation cephalosporins reached 60% and 18%, respectively, in Myanmar [19,20]. Sixty percent of *Acinetobacter* species, 60% of *E. coli*, 55% of *Klebsiella* species, 60% of *Pseudomonas* species, and 36% of *Staphylococcus* species were resistant to GM at North Okkalapa General Hospital in Myanmar [21]. Quality of antimicrobials is a key issue to prevent emergence of antimicrobial resistance.

We found that 3 GM samples were counterfeit, containing little or no potency of GM, and would likely be ineffective, and rather contribute to bacterial resistance. In order to identify counterfeit medicines efficiently, it is indispensable to get more cooperation from manufacturers, and governments of manufacturing countries, in responding to our questionnaire for authenticity investigation.

In the cases of CTRX and DN, the results were satisfactory.

Regarding CXM, two-thirds of samples passed the quality tests, while one-third proceeded to the 2nd stage tests or permanently failed because of relatively small deviations from tolerance. Half of the latter samples were manufactured by an Indian company, though some products from almost all manufacturers slightly fell below the required standards. 

In the case of OM, at least 33.8% of samples failed the DS in the acid phase and/or buffer phase. This is similar to the high failure ratio in DS of OM samples collected in the Cambodian pharmaceutical market [4,22]. The cause of this high DS failure ratio of OM in Myanmar should be investigated. Most failed OM samples were manufactured by companies in India, though some products from almost all manufacturers slightly fell below the required standards.

Storage conditions in Yangon were not necessarily favorable and might be a contributory factor to the failures of medicines in quality tests. Although CXM, DN, OM, CTRX, and GM should be stored below 25 °C or 30 °C in a dry place, and should be protected from light and moisture, according to the labeled storage information [22,23,24], an air-conditioner was operating in fewer than half of the retail shops. To obtain better quality medicines, it will be important to improve the storage conditions at drug outlets. Indeed, in our previous study, most of the OM samples failed the quantity tests, as well as the dissolution tests [22].

The prices of all-exam-passed CXM were significantly higher than those of other samples, whereas there appear to be no difference in the case of CTRX. Counterfeit GM tended to be cheaper than good-quality GM. Lower prices may be one of the clues to find poor-quality medicines, though further investigation of this point will be necessary to get reliable answers. 

The current study has some limitations. In particular, the region of sample collection was limited, sample size was also limited, and random sampling would have been desirable. Consequently, our findings may not accurately reflect the overall situation in Myanmar. Also, in authenticity investigation, the response rate from the manufacturers was quite low, and unsurprisingly, manufacturers who produced counterfeit medicines never answered.

## 5. Conclusions

Counterfeit GM was sold in Yangon. A substandard concentration of the active ingredient was the main reason for low quality, which can lead to increased morbidity and mortality and the emergence of antimicrobial resistance. Furthermore, qualities of some CTRX, CXM, and OM were poor, and there were some unregistered GM and DN. Regular surveys to monitor counterfeit and substandard medicines in Myanmar are recommended.

## Figures and Tables

**Table 1:** The number of collected samples.

Items	Dosage Form	Government Hospital	Private Hospital	Community Pharmacies	Clinical Pharmacies	Wholesalers
CXM (n = 60)	Oral medicine	14	12	22	9	3
DN (n = 3)		0	0	2	0	1
OM (n = 65)		13	12	30	6	4
CTRX (n = 49)	Injection	9	11	18	7	4
GM (n = 58)		11	7	31	6	3
Total (n = 235)		47 (20%)	42 (17.9%)	103 (43.8%)	28 (11.9%)	15 (6.4%)

CXM: cefuroxime, DN: donepezil, OM: omeprazole, CTRX: ceftriaxone, GM: gentamicin.

**Table 2:** Investigated samples.

Active Ingredient	CXM	DN	OM	CTRX	GM	Total (%)
Strength and Dosage Form	Tablet 250 mg/n = 59 (1) Tablet 500 mg/n = 1	Tablet 5 mg/n = 3	Capsule 20 mg/n = 63 Tablet 20 mg/n = 2	Vial 1g/n = 49	Ampule 80 mg/n = 58	
Number of samples collected	60 (1)*	3	65	49	58	235 (100)
Bangladesh	2		2	2	4	10 (4.3)
China				1	16	17 (7.2)
India	44/1 **	1	57/2 ***	39	5	149 (63.4)
Japan		2				2 (0.9)
Korea				2	4	6 (2.6)
Myanmar				3	11	14 (6.0)
Pakistan	1			1		2 (0.9)
Singapore			1			1 (0.4)
Switzerland				1		1 (0.4)
Taiwan					14	14 (6.0)
Thailand			3			3 (1.3)
UK	11					11 (4.7)
Vietnam	1				4	5 (2.1)
Number of manufacturers	12	2	21	19	17	71 (100)
Bangladesh	2		1	2	1	6 (8.5)
China				1	7	8 (11.3)
India	6/1 **	1	17/1 ***	12	2	40 (56.3)
Japan		1				1 (1.4)
Korea				1	1	2 (2.8)
Myanmar				1	3	4 (5.6)
Pakistan	1			1		2 (2.8)
Singapore			1			1 (1.4)
Switzerland				1		1 (1.4)
Taiwan					1	1 (1.4)
Thailand			1			1 (1.4)
UK	1					1 (1.4)
Vietnam	1				2	3 (4.2)
Number of products	12	2	21	19	17	71

CXM: cefuroxime, DN: donepezil, OM: omeprazole, CTRX: ceftriaxone, GM: gentamicin. ()* Number of samples among the total collected number of samples for which the amount was too small to conduct full scale of pharmacopoeial testing, and only authenticity investigation and limited quality tests were implemented. ** Fourty four 250 mg tables and one 500 mg tablet. *** Fifty-seven capsules and one tablet.

**Table 3:** MFDA registration of each medicine.

Items	Dosage Form	Samples Registered by MFDA	Samples Unregistered by MFDA
CXM (n = 60)	Oral medicine	60	0
DN (n = 3)		2	1
OM (n = 65)		65	0
CTRX (n = 49)	Injection	49	0
GM (n = 58)		53	5
Total (n = 235)		229 (97.4%)	6 (2.6%)

CXM: cefuroxime, DN: donepezil, OM: omeprazole, CTRX: ceftriaxone, GM: gentamicin, MFDA: Food and Drug Administration, Myanmar.

**Table 4:** The number of samples that passed each quality analysis test.

Items	Dosage Form	Quantity Test	Content Uniformity Test	Dissolution Test	Endotoxin Test	Sterility	Identification	Microbial Assay
CXM (n = 60)	Oral medicine	49/60 (81.7)	44/60 (73.3)	56/60 (93.3)	-	-	-	-
DN (n = 3)		3/3 (100)	3/3 (100)	3/3 (100)	-	-	-	-
OM (n = 65)		42/65 (64.6)	56/65 (86.2)	48/65 (73.8)	-	-	-	-
CTRX (n = 49)	Injection	47/49 (95.9)	46/49 (93.9)	-	49/49 (100)	49/49 (100)	-	-
GM (n = 58)		-	-	-	58/58 (100)	58/58 (100)	55/58 (94.8)	55/58 (94.8)
Total (n = 235)		141/177 (79.7)	149/176 (84.7)	105/128 (82.0)	107/107 (100)	107/107 (100)	55/58 (94.8)	55/58 (94.8)

Expressed the number of all-exam-passed sample/tested samples (%); CXM: cefuroxime, DN: donepezil, OM: omeprazole, CTRX: ceftriaxone, GM: gentamicin.

**Table 5:** Prices differences by medical quality.

Items	Test Results	n	Mean (Kyat) ± S.D.	Differences
CXM	Passed all exams	44	654.9 ± 206.54	*p* < 0.05
	Others	16	368.9 ± 120.78	
DN	Passed all exams	3	3248.7 ± 1230.42	*
	Others	0	-	
OM	Passed all exams	32	49.0 ± 30.47	n.s.
	Others	33	49.8 ± 32.55	
CTRX	Passed all exams	46	1634.1 ± 1039.24	*
	Others	3	1650.0 ± 650.00	
GM	Uncounterfeit	55	145.1 ± 72.98	*
	Counterfeit	3	38.3 ± 10.41	

CXM: cefuroxime, DN: donepezil, OM: omeprazole, CTRX: ceftriaxone, GM: gentamicin; Prices were compared between good quality ampoules and poor-quality ones using *t* test. n.s.: not significant; * Statistically uncalculated due to few or no samples.
